# A preclinical mice model of multiple sclerosis based on the toxin-induced double-site demyelination of callosal and cerebellar fibers

**DOI:** 10.1186/s40659-024-00529-7

**Published:** 2024-07-22

**Authors:** Sebastián Vejar, Ignacio S. Pizarro, Raúl Pulgar-Sepúlveda, Sinay C. Vicencio, Andrés Polit, Cristian A. Amador, Rodrigo del Rio, Rodrigo Varas, Juan A. Orellana, Fernando C. Ortiz

**Affiliations:** 1 Mechanisms of Myelin Formation and Repair Laboratory, Departamento de Biología, Facultad de Química y Biología, Universidad de Santiago de Chile, Santiago, Chile; 2 Faculty of Medicine and Science, Universidad San Sebastián, Santiago, Chile; 3 Laboratory of Cardiorespiratory Control, Department of Physiology, Pontificia Universidad Católica de Chile, Santiago, Chile; 4 Department of Cell Biology and Physiology, School of Medicine, University of Kansas Medical Center, Kansas City, KS United States; 5 Facultad de Ciencias de Salud, Universidad Autónoma de Chile, 8910060 Santiago, Chile; 6 Departamento de Neurología, Escuela de Medicina and Centro Interdisciplinario de Neurociencias, Facultad de Medicina, Pontificia Universidad Católica de Chile, Marcoleta 391, 8330024 Santiago, Chile

**Keywords:** Myelin, Multiple sclerosis, White matter, Neurodegeneration, Neuroinflammation

## Abstract

**Background:**

Multiple sclerosis (MS) is an irreversible progressive CNS pathology characterized by the loss of myelin (i.e. demyelination). The lack of myelin is followed by a progressive neurodegeneration triggering symptoms as diverse as fatigue, motor, locomotor and sensory impairments and/or bladder, cardiac and respiratory dysfunction. Even though there are more than fourteen approved treatments for reducing MS progression, there are still no cure for the disease. Thus, MS research is a very active field and therefore we count with different experimental animal models for studying mechanisms of demyelination and myelin repair, however, we still lack a preclinical MS model assembling demyelination mechanisms with relevant clinical-like signs.

**Results:**

Here, by inducing the simultaneous demyelination of both callosal and cerebellar white matter fibers by the double-site injection of lysolecithin (LPC), we were able to reproduce CNS demyelination, astrocyte recruitment and increases levels of proinflammatory cytokines levels along with motor, locomotor and urinary impairment, as well as cardiac and respiratory dysfunction, in the same animal model. Single site LPC-injections either in corpus callosum or cerebellum only, fails in to reproduce such a complete range of MS-like signs.

**Conclusion:**

We here report that the double-site LPC injections treatment evoke a complex MS-like mice model. We hope that this experimental approach will help to deepen our knowledge about the mechanisms of demyelinated diseases such as MS.

## Introduction

Multiple sclerosis (MS) is an irreversible, progressive disease characterized by an autoimmune attack of T and B lymphocytes against self-antigens found in the myelin, a specialized membrane that enwraps axons [26, 41]. The myelin enables axons for fast saltatory conduction of action potentials and provides metabolic support to the neurons [11, 31]. The lack of myelin leads, in turn, to axonal degeneration, neuronal death, and several neurological disabilities [11, 38]. MS affects around 2.5 million people worldwide, representing the second cause of neurological impairments in the young adult population [30].

The demyelinated insult is followed by a spontaneous, yet incomplete, myelin repair process (i.e. remyelination) [26]. In this microenvironment characterized by neuroinflammatory signals, oligodendrocytes—the myelin forming cells at the central nervous system (CNS)—fail to synthesize new myelin [19, 43]. The latter leads to progressive axon degeneration, aggravating the neurological symptoms in MS patients, such as motor and sensory impairment, fatigue along with locomotor, cardiac, respiratory and bladder dysfunctions [6, 11, 21, 31, 38]. Currently, there are more than fourteen approved disease modifying therapies (DMT) to treat MS, including anti-inflammatory drugs, immunomodulators (i.e. interferons) and more recently, monoclonal antibodies (such as alemtuzumab or ocrelizumab). Most of them aim to contain inflammation, reducing relapses and delaying the progression of the disease [7]. However, nowadays, there are no DMT capable of inducing complete recovery of the patient [30]. Therefore, preclinical studies including animal models are currently being carried out to address potential mechanisms that lead to new treatments [13, 42]. In this line, the experimental autoimmune encephalomyelitis (EAE) murine model represents the primary experimental approach for studying inflammatory demyelinating diseases like MS [5, 42]. Nevertheless, some authors argue that EAE might be better suited as a neuroinflammatory model rather than a MS model [27]. EAE animals exhibit inflammatory and cellular responses like those observed in MS patients, along with progressive motor function impairment as a major clinical-like outcome. One-site injection of demyelinated toxins (i.e. lysolecithin (LPC) or ethidium) in small volumes (0.1–0.5 µL) into areas like the corpus callosum or cerebellar white matter [14, 32, 37] or cuprizone administered in the water or food of animals (0.2–0.3%p/p) [24, 28], recapitulates various aspects of the cellular response and neuroinflammation. LPC has been used as demyelinating agent during decades [18]. Most recent evidence suggests that exogenous injections of LPC would impair an endogenous LPC-buffering system that controls myelin-lipid homeostasis, triggering a non-specific disruption of it, leading to oligodendrocytes dead with the consequent myelin loss [34]. However, LPC fails to correlate with pre-clinical outcomes typically seen in MS [32, 42]. Thus, current preclinical models of MS can replicate cellular aspects of the disease and some clinical signs, commonly associated with motor function. Nevertheless, they do not reproduce a diverse range of cellular and clinical outcomes necessary for a more comprehensive pre-clinical model of MS [27, 42]. Here, we show that by injecting LPC in both callosal and cerebellar white matter fibers of mice, we develop CNS neuroinflammatory demyelination along with locomotor, cardiac, respiratory and bladder dysfunctions.

## Materials and methods

### Reagents and antibodies

Lysolecithin (LPC), ketamine, xylazine, Fluoromount-G mounting medium, paraformaldehyde (PFA), normal goat serum, Triton X-100, anti-MBP chicken polyclonal antibody (Thermofisher). Anti-GFAP mouse monoclonal antibody, HEPES, Alexa dye secondary antibodies AF-488 and AF-633 were purchased from Sigma-Aldrich (Merck Group, St. Louis, MO, USA). Anti-TNF-α mouse monoclonal antibody (Santa Cruz Biotechnology). The diamidino-2-phenylindole (DAPI), probenecid (PBC), and goat anti-mouse Alexa Fluor 488) were obtained from Thermofisher (Waltham, MA, USA).

### Animals

**Fig. 1:**
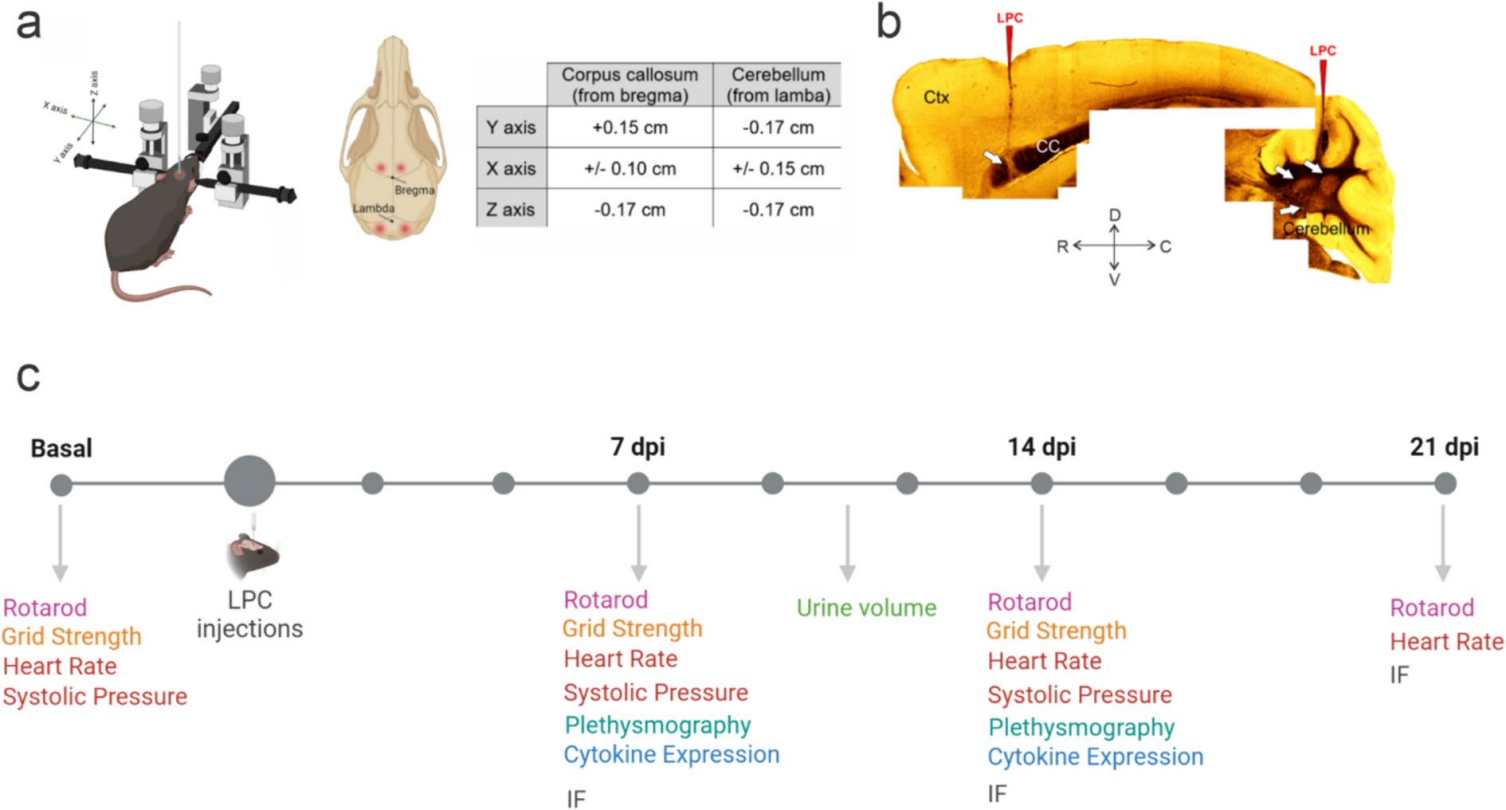
Model of double-site LPC injection into the corpus callosum and cerebellar fibers. **A** Schematics of the stereotaxic set up for the microinjection of LPC showing the sites of LPC injections in corpus callosum and cerebellar white matter. Note the different references for callosal (bregma) versus cerebellar (lambda) sites and the specific coordinates depicted in the table. **B** Reconstitution of several bright fields (no staining) of a representative mice subjected to double-site LPC injection in corpus callosum and cerebellar fibers (right panel). Red arrows indicate the sites of injection (note the path of graphite powder left by the pipette during the surgery, see methods), while white arrows indicate focal points of demyelinated lesions at 7 days post injection (dpi). *Ctx* cortex, *CC* corpus callosum. R, C, D, V indicates rostral, caudal, dorsal and ventral, respectively. **C** Overview of the experimental design. Two independent experimental groups were randomly destined to LPC or PBS double-site LPC injections. Locomotor (grid strength and Rotarod tests), cardiac (heart rate test) and ventilatory (plethysmography test) functions were previously applied to assess basal levels. *IF* immunofluorescence. For details, see “Materials and methods” section

Animal experimentation and protocols were approved by the Bioethical Committee for Animal Experiments of the University of Santiago de Chile (protocol number 319/2023) in accordance with Guide for the Care and Use of Laboratory Animals (National Institutes of Health, USA). C57BL/6 mice (PN45-60) from the animal facility of the University of Santiago de Chile were housed in cages in a temperature-controlled (24 °C) and humidity-controlled vivarium under a 12 h light/dark cycle (lights on 8:00 a.m.), with ad libitum access to food and water. Animals were daily checked by the faculty veterinarian assuring mice welfare. The experimental design is summarized in Fig. 1A–C.

### Double-site focal demyelination by lysolecithin (LPC) injections

Mice were anesthetized with ketamine/xylazine (K/X, 0.1/0.01 mg/g). Deeply anesthetized mice (approximately 15 min after K/X administration) were gently fixed in a stereotaxic surgery frame (Kopf Instruments, CA, USA), fitting its head parallel to the base. An embedded towel paper with ethanol 70% was used to sterilize the surgery zone. Mice hair was removed from the upper skull using a cotton swab and commercial shaving cream was applied. Shaving solution was removed and fully wiped out with a water embedded cotton swab 1 min after exposure to avoid skin irritation. Any remaining hair was removed as well. A drop of PBS 1× was added over each mice eye to avoid drying damage. Mice were treated with a carprofen solution (5 mg/kg) as analgesia (to keep mice hydrated during surgery, small PBS volumes ~ 100 µL were added subcutaneously). Then, a clean incision was made in the head skin with surgery scissors. Meninges were carefully removed with cotton fibers to provide clean access to the skull bone. To target the corpus callosum we used the following coordinates antero-posterior + 1.5 mm, medio-lateral ± 1 mm, dorso-ventral − 1.7 mm from bregma [32, 37], and to target cerebellar white matter antero-posterior + 1.7 mm, medio-lateral ± 1.5 mm, dorso-ventral − 1.7 mm from Lamba (note that the injections are bilaterally applied, as shown in Fig. 1). The craniotomy to access the brain surface at the right coordinates was performed with a Micromotor High-Speed Drill (Stoelting C., IL, USA). Then, we proceed with the intracranial injections of 2 μL of 2% LPC solution per injection site. Injections were achieved by using a Hamilton syringes (10 μL) connected to a pulled-glass pipette (any conventional capillary can be used) previously loaded with the LPC solution (2% w/v diluted in PBS solution, vortex if needed). To identify the lesion sites afterwards, we embedded the tip of the pipette with sterile graphite powder (see Fig. 1). The glass micropipette was placed right above the drill incision and stereotaxically downplaced in the corresponding Z-axis coordinate. After 3 min, the LPC solution was carefully injected 0.3–0.5 μL at a time, with a 3-min pause between injections until 2 μL (for callosal fibers) or 2.5 µL (for cerebellum) was reached (2% LPC solution in both regions). Then, another 3-min pause was made. Finally, the micropipette was carefully and slowly removed back from the mouse brain. The procedure was repeated in the contralateral hemisphere in both callosal and cerebellar regions (to achieved a total of 4 injection sites, Fig. 1). Mice in the control group were injected with vehicle (PBS) in a separate experimental group. Finally, open skin was closed with a sterile size #2 suture. After recovery, mice were treated with analgesia for 2 days (carprofen, s.c. 5 mg/kg) and checked each day following the MGS according to the experimental protocols approved by the Bioethical Committee for Animal Experiments of the Universidad de Santiago de Chile (protocol number 319/2023).

Myelin protein expression—as a measurement of (de)myelinated area—was assessed 7, 14 or 21 days after LPC injections (dpi). From a total of 42 injected mice, 4 animals died during the first week after LPC or PBS injections, and 3 mice were excluded from the study due to the lack of demyelinated areas and/or the display of mechanical lesions (see also [32]). All the animals considered in the study (n = 35) survive the entire experimental time.

### Tissue preparation

Mice were anesthetized using ketamine/xylazine (0.1/0.01 mg/g) and subsequently subjected to intracardial perfusion with a 20 mL PBS 1× solution (pH 7.2). Following perfusion, the brains were carefully extracted to maintain their structural integrity. A precise transversal cut was made at the midline, leaving one hemisphere stored in a 4% PFA solution (pH 7.2) for 3 h at 4 °C, while the other half was prepared for molecular analysis and kept at − 80 °C. Once fixed, the brain tissue was sectioned into 70 µm sagittal slices using a vibratome (Microm HM 650 V, Thermo) and these slices were then placed in 96-well plates filled with 1 mL PBS 1×. Slices containing the corpus callosum and cerebellar regions were specifically chosen for subsequent immunofluorescence assays.

### Immunofluorescence

For immunofluorescence analysis, the slices were incubated in blocking solution (Normal goat serum 4% and Triton X-100 0.5% in PBS 1×) at room temperature for 2 h. The sections were then incubated overnight at 4 °C in the antibody solution (Normal goat serum 2% and Triton X-100 0.2% in PBS 1×) with the myelin basic protein (anti-MBP chicken polyclonal antibody, 1:800, #PA1-10008) or the glial fibrillary acidic protein (anti-GFAP mouse monoclonal antibody, 1:2000, #SAB5201104). The slices were three times rinsed in PBS 1× (5 min each) and then incubated during 2 h at room temperature with conjugated Alexa dye secondary antibody AF-488 or AF-633 (goat anti-chicken, 1:500; Invitrogen, #A-11039). Specificity of the anti-MBP was verified in negative controls, omitting the primary antibodies. Finally, slices were washed three times with PBS 1× and mounted in Fluoromount-G mounting medium (#0100-01, SouthernBiotech) for imaging.

### Confocal acquisition

Brain slices were imaged with a Zeiss LSM510 confocal microscope (Carl Zeiss MicroImaging) with the LSM510 software. Bright field images (transmitted light, see Fig. 1C) were acquired to define corpus callosum and cerebellar white matter regions. Images were then captured with a 20× (NA 0.8) objective under 488 nm excitation in Z-stack 6 µm-width. Acquired images were then reconstructed in Z-projections averaging 8 to 12 optical sections per sample (48 to 72 µm-width). Image analysis (MBP and GFAP fraction areas) was performed with ImageJ software.

### Cytokine expression

To analyse cytokine gene expression we performed a three phases analysis: total RNA extraction, cDNA synthesis, and quantification via qPCR. For RNA extraction from white matter tissue (isolated cerebellar white matter and corpus callosum), we employed the TRIzol-based method optimized for this purpose. Samples stored at − 80 °C were homogenized by using 1.0 mL of TRIzol with mechanical homogenizers (D160 SCILOGEX) and/or homogenization equipment (ALLSHENG Bioprep-24R). Phase separation was achieved by adding 200 µL of chloroform, followed by centrifugation. The recovered aqueous phase was precipitated with isopropanol and washed with 75% ethanol. Purified RNA was resuspended in RNase-free water and stored at − 80 °C. From 20 µg of total RNA, cDNA synthesis was performed using the M-MLV system from Promega. DNase treatment was applied to remove potential DNA contaminants, followed by addition of Random Primer to facilitate the cDNA synthesis. The reaction involved three stages: DNase treatment, annealing of random primers, and synthesis of the first cDNA strand. The reaction was first incubated at 37 °C for 60 min and then at 70 °C for 10 min to complete the synthesis. Gene expression quantification was conducted using FastStart Essential DNA Green Master (ROCHE®). The reaction mix, comprising 2× Master mix, forward and reverse primers (10 µM), water, and cDNA, was prepared (max 200 ng cDNA per reaction). Amplification was performed in a thermocycler programmed for an initial polymerase activation stage at 95 °C for 10 min, followed by 40 cycles of denaturation at 95 °C, annealing at 62 °C, and extension/reading at 72 °C. A melting curve analysis was conducted post-amplification to confirm the specificity of the products. Relative gene expression was determined using the \ΔΔC_T\ method, normalized against the reference gene 18S and compared to an untreated control. The efficiency of the qPCR reaction was validated through the generation of standard curves for each gene of interest. Additionally, we performed western blot quantification of TNF-α. Briefly, white matter was homogenized in 10 mM Tris–HCl at pH 7.4, 150 mM NaCl, 1% Triton-X 100, and 1 mM ethylenediaminetetraacetic acid (EDTA) containing protease inhibitors. Lysates were clarified by centrifugation at 8000×*g* (4 °C) for 20 min, and the supernatants were collected and normalized for protein concentration. Proteins were separated by 10% and 15% sodium dodecyl sulfate-polyacrylamide gel electrophoresis (SDS-PAGE) and transferred onto polyvinylidene difluoride membranes (Immobilon-P, Millipore). After blocking with PBS containing 5% skim milk and 0.05% Tween 20, the membranes were incubated with primary antibodies overnight at 4 °C, followed by incubation with an HRP conjugated secondary antibody for 2 h at room temperature. Mouse anti-TNF-α (1:500, sc-133192). Horseradish peroxidase-conjugated anti-rabbit IgG antibody was used as secondary antibody (Cell Signaling Technology). Immunoreactive bands were detected using a fluorescence-conjugated secondary antibody and an enhanced chemiluminescence (ECL) system (WBKLS0100, Millipore). They were visualized on a LAS-4000 imaging system (Fujifilm). The protein bands were quantified using the ImageJ software.

### Plethysmography

Whole-body plethysmography was performed in freely moving mice to evaluate their resting breathing patterns and chemoreflex function in response to hypercapnia. The experiments were carried out between 10:00 and 16:00 h at room temperature. Prior to recordings, animals were habituated to a whole-body plethysmography chamber (5L, EMKA Technologies) for 2 consecutive days. On the day of recording, mice were allowed a minimum of 2 h acclimatization to the chamber before measurement started. Respiratory flow was recorded using a differential pressure transducer, with the signal being amplified (500×) and digitized at 1 kHz. The inspiratory flow curve’s area was calibrated by injecting 5 mL of dry air into the chamber using a syringe. Throughout the 2-h recordings, mice breathed ambient air with constant inlet and outlet flows (0.75 mL/min). Chemoreflex function was examined by exposing the mice to hypercapnic concentrations of 3%, 5%, and 7%, while recording the ventilatory response and calculating the hypercapnic ventilatory response (HCVR). Data analysis involved acquiring tidal volume (Vt), and minute ventilation (V_E_) using the EMKA software. The hypercapnic ventilatory response (HCVR) was determined by calculating the slope of the maximal ventilatory response across different CO_2_ levels.

### Grid strength test

The grid strength test assesses neuromuscular strength by measuring how long a mouse can hang from an inverted grid [3, 23, 25]. Briefly, mice were gently placed on a 30 × 30 cm grid with 1 cm^2^ mesh spaces, positioned 30 cm above a plastic containment box. The grid was carefully rotated so that the mouse faced downward. The time it took the mouse to fall (time-to-fall) was recorded. This measurement was repeated twice for each mouse, and the averaged time was registered. If a mouse jumped or climbed down the box edges, the count was stopped. After a 5-min break, the test was repeated. If a mouse hung for 600 s without falling, the test was ended, and that time was recorded as the maximum value. Mice were allowed 3 days of acclimatization to the apparatus before starting the recordings. Time-to-fall was measured on day-2 before LPC injection and then on days 5, 6, and 7 post-injection. The same mouse was tested at all time points, and then tested in the locomotor Rotarod test. Results were expressed in seconds. All injected animals (n = 4) developed an impairment in the test.

### Locomotor Rotarod test

The Rotarod test was employed to assess locomotor performance, evaluating motor coordination and balance [39]. The Rotarod apparatus consists of cylinders (or rotors) with individual lanes that increase in turning speed. Rodents are placed in these lanes at secure height (Rotarod 755, IITC, Lifescience). Before beginning the recordings, animals underwent a daily acclimatization session in the apparatus for 3 days. Time-to-fall on the Rotarod was measured 5 days before LPC injection (− 5 dpi) and then at 7, 14 and 21 days post injection (dpi). During training sessions, parameters included a starting speed of 1 rpm, a maximal speed of 40 rpm and a time to reach maximal speed of 90 s. Experimental conditions consisted of a starting speed of 1 rpm, a maximal speed of 40 rpm and a time to reach maximal speed of 180 s. The same animal was tested at all time points and time-to-fall was expressed in seconds.

### Urine volume

To assess the diuretic function, we used metabolic cages to collect and quantify urine volume as an indicator of uronephrological or bladder status [16]. Mice underwent testing in individual metabolic cages for 12-h sessions at night, from 8 p.m. to 8 a.m., with environment conditions set at 20–22 °C and 50% humidity. This regime was repeated for 4 consecutive days. During testing, mice has access to water and previously ground food ad libitum, and each mouse remained in the same cage throughout all sessions. The first three sessions served for mouse acclimatization, with urine volume recorded on the last day. Assessments were conducted both before model induction (basal) and at days 8–11 post LPC (or vehicle) injection.

### Systolic pressure and heart rate measurement

Systolic pressure and heart rate (HR) were measured in awake mice using a tail cuff plethysmographic system, (model BP-2000 series II, Visitech, US) as described previously [2, 9]. Briefly, mice underwent a 3-day training period prior to the recording session, maintaining consistent environmental parameters: room temperature of 20–22 °C, 50% humidity, and a tail-cuff system temperature of 37 °C. During the recording session, mice were placed in a black box to restrict movement and their tails were secured with adhesive tape to facilitate pulse measurement at the base of the tail. Every session included a 15-min acclimatization period, during which three consecutive readings were not recorded, followed by 10 consecutive readings of arterial pulsatile and mean pressure captured by the BP—2000 Blood Pressure Analysis Software. Assessments were conducted before model induction (basal, − 5 dpi) and at days 7, 14 and 21 days post LPC injection.

### Statistical analysis

Statistical analysis and plots were performed using Graphpad Prism 8.0 software. All data is presented as mean ± SEM. After test for normality, data were compared by using One- or Two-way ANOVA, Kruskal Wallis, or Mann Whitney Tests according to the data structure. Post hoc test is indicated when corresponded. Statistical significance was defined as p < 0.05.

## Results

### Double-site LPC injection into callosal and cerebellar white matter fibers induces demyelination, pro-inflammatory cytokine expression and astrocyte recruitment

**Fig. 2:**
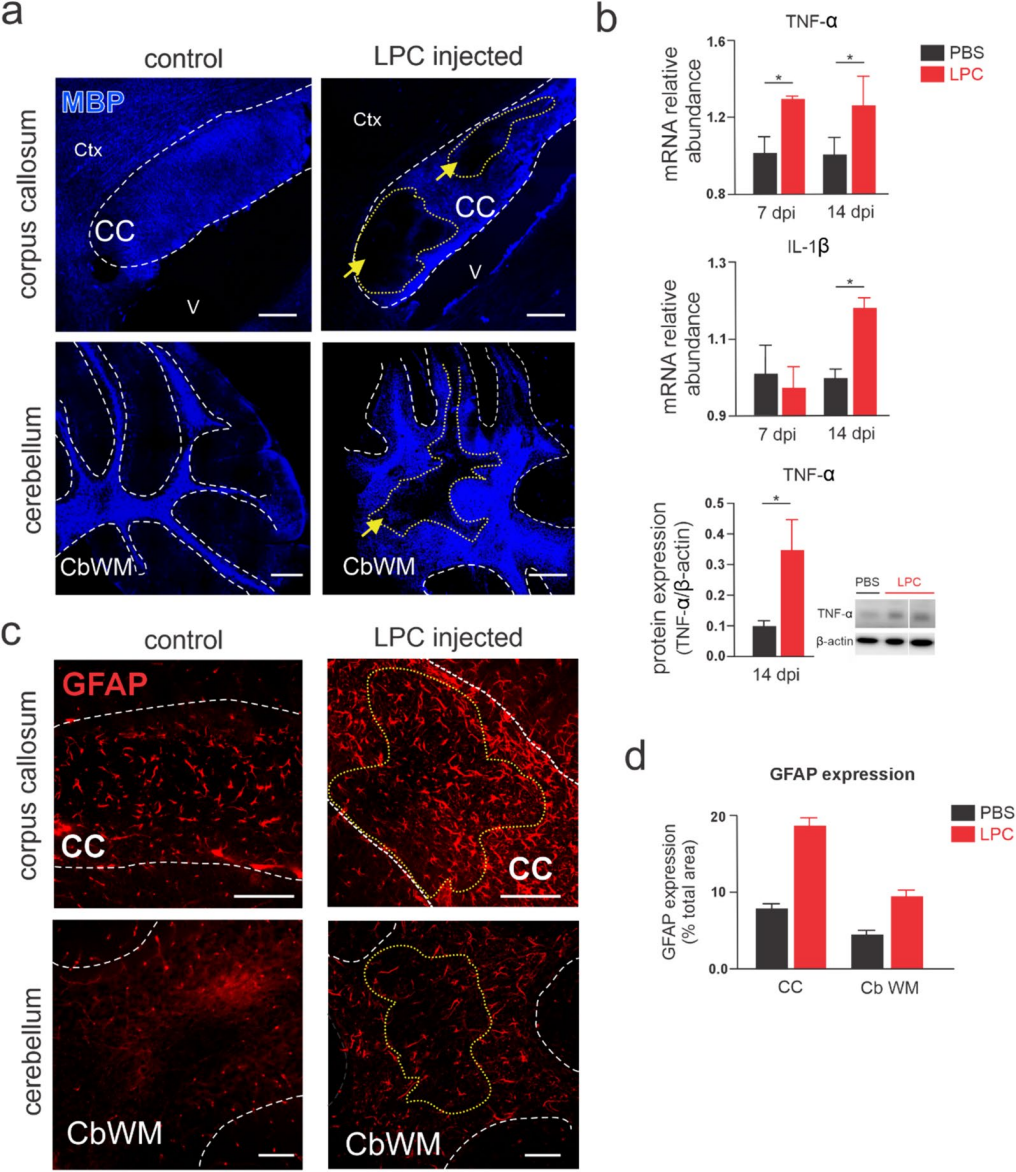
Double-site LPC injection into callosal and cerebellar fibers induces localized myelin loss, increased cytokine expression, and astrocyte recruitment. **A** Representative confocal images depicting MBP staining (blue) in callosal and cerebellar regions (upper and lower panel respectively, sagittal views) by mice under control conditions (left) and after 7 days of LPC injection (right). Note the lack of myelin (MBP labeling, yellow arrows/demarked) in LPC-treated animals compared with the expression of MBP observed in control conditions (n = 5–6 mice per condition, from a total of n = 7–8 injected animals). *Ctx* cortex, *CC* corpus callosum, *St* striatum, *CbWM* cerebellar white matter. Scale bars: 150 μm. **B** Averaged data of TNF-ɑ (upper) and IL-1β (middle) mRNA expression in demyelinated white matter (see Materials and methods) by mice following 7 or 14 days post-injection (dpi) with PBS (black bars) or LPC (red bars) in the corpus callosum and cerebellar white matter. Protein levels of TNF-α (normalized by β-actin, bottom) at 14 dpi are also shown (representative images of western blot membranes from two double-site LPC injected mice). *p < 0.05, LPC treatment compared to control (PBS) conditions (Kruskal–Wallis test followed by a Dunns multiple comparison post hoc, n = 3 mice per condition). **C** Representative confocal images depicting GFAP staining (red) in callosal and cerebellar regions (upper and lower panel respectively, sagittal views) by mice under control conditions (left) and after 7 days of LPC injection (right). Note the increase of the GFAP stained area around or in the lesioned regions (yellow arrows/demarked) in LPC-treated animals compared with the expression of GFAP in control conditions (n = 2–3 mice per condition). Scale bars: 100 μm. **D** Averaged data of GFAP expression (percentage from the total region) by mice at 7 days post-injection (dpi) with PBS (black bars) or double-site LPC injections (red bars) in the corpus callosum (CC) and cerebellar white matter (CbWM) (n = 2–3 mice per condition)

**Fig. 3:**
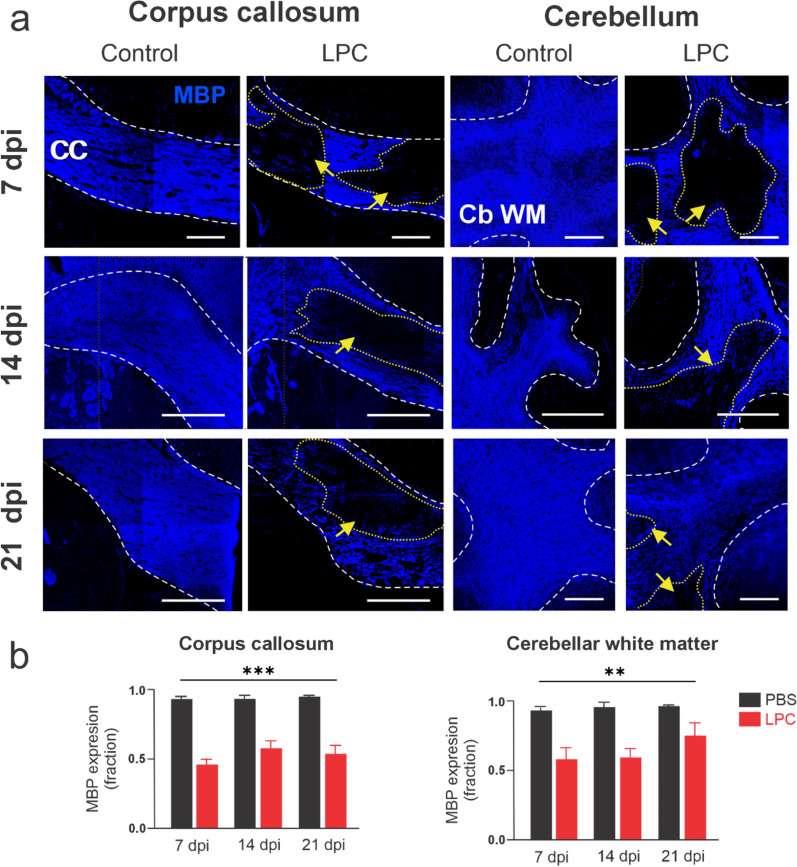
Double-site LPC injection into callosal and cerebellar fibers induces localized myelin loss over time. **A** Representative confocal images depicting MBP staining (blue) in callosal and cerebellar regions (left and right panels respectively) over time at 7, 14 and 21 days after double-site LPC injections (dpi). Note the lack of myelin (MBP labeling, yellow arrows/demarked) in LPC-treated animals compared with the expression of MBP observed in control conditions (n = 5–6 mice per condition). Scale bars: 200 μm. **B** Averaged data of MBP expression (fraction from the total region) by mice following 7, 14 or 21 days post-injection (dpi) with PBS (black bars) or LPC (red bars) in the corpus callosum and cerebellar white matter. ***p < 0.001, **p < 0.01, LPC treatment compared to control (PBS), Two-way ANOVA mixed effect analysis

Injection of LPC into white matter tracts induces demyelination [18, 22, 33, 45], mimicking the effects observed in demyelinating diseases of CNS [10, 20]. Even though one-site LPC injection in the corpus callosum or cerebellar white matter recapitulates different aspects of the cellular response and neuroinflammation, it fails to correlate with pre-clinical outcomes of MS [14, 32, 37, 42]. With this in mind, we aimed to investigate whether the bilateral injection of LPC into both the corpus callosum and cerebellar white matter in the same surgery induces a scenario that better resemble the pre-clinical outcomes of MS. For this purpose, we stereotaxically administered 2% LPC in focal areas of both white matter tracts (Fig. 1A, B). Immunohistochemistry analysis of MBP labeling showed significant demyelination in both callosal and cerebellar white matter fibers 7, 14 and 21 days after the double-site LPC injection (Figs. 2A, B, 3A, B). Previous evidence indicates that one-site LPC injection in the corpus callosum or cerebellar white matter produces local inflammation [37, 42], including the increased production of pro-inflammatory cytokines such as TNF-α and IL-1β [40, 42, 46]. Accordingly, we examined the expression of both cytokines in our system. Quantitative PCR analysis showed that LPC led to a two-fold increase in TNF-α expression 7 days post-injection, and this effect persisted for at least 14 days after LPC administration (Fig. 2B). In addition TNF-α protein levels were three times higher at 14 dpi (Fig. 2B). The expression of IL-1β increased only after 14 days post-LPC injection when compared to control conditions (Fig. 2B). Another important hallmark of demyelinated lesions is the recruitment of glial cells that contribute to the neuroinflammatory microenvironment [12, 43]. Accordingly, we found an increased expression of the astrocyte marker GFAP in demyelinated areas at 7 dpi (Fig. 2C, D) suggesting the recruitment of astrocytes to the lesions as it have been consistently described [12, 14, 29, 43]. Altogether, these findings indicate that double-site LPC injections of callosal and cerebellar white matter fibers trigger demyelination and increased the recruitment of astrocytes along with the augmented expression of TNF-α and IL-1β.

### Double-site callosal and cerebellar demyelination causes major motor dysfunction

**Fig. 4:**
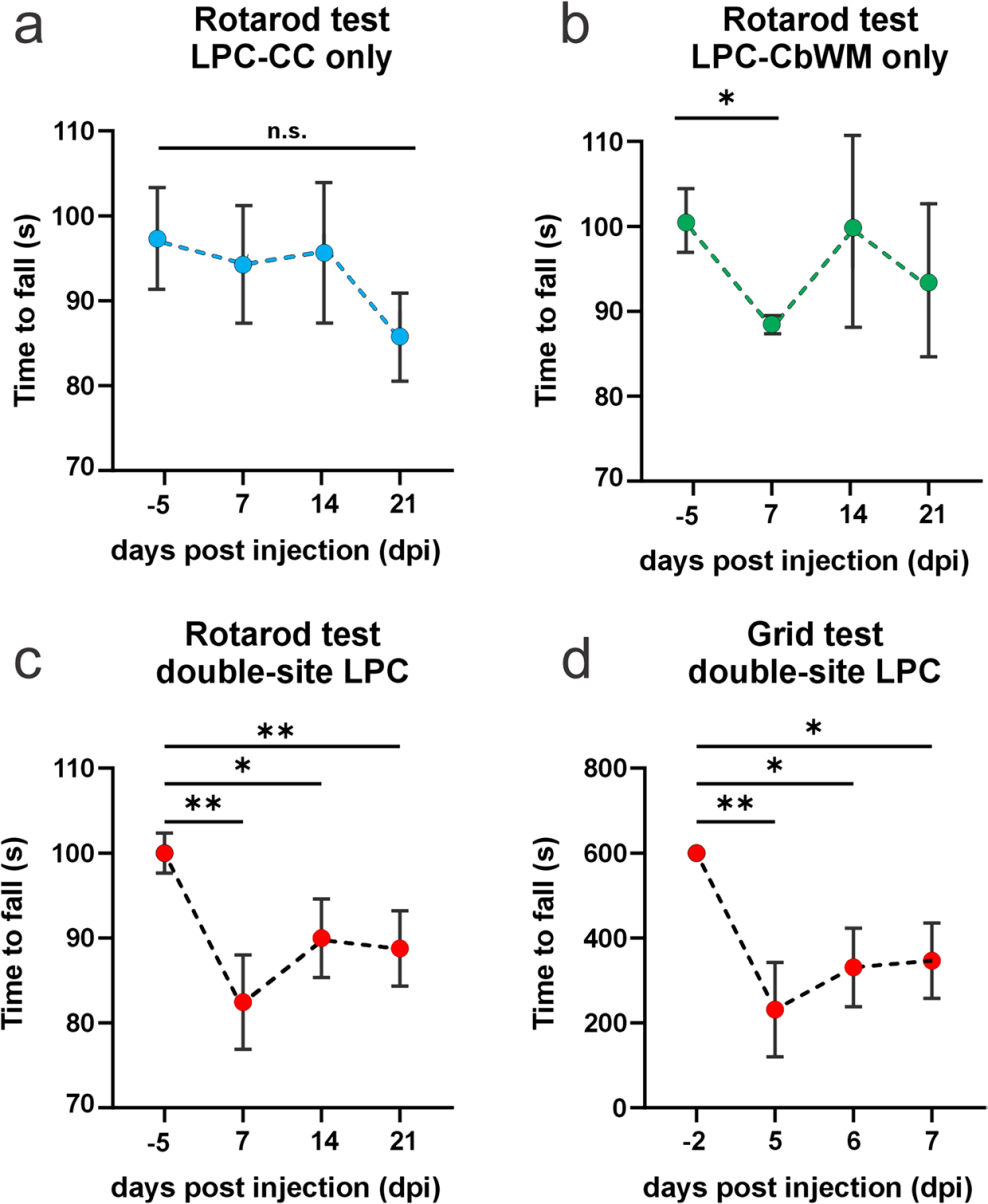
Double-site LPC injection into callosal and cerebellar white matter fibers impairs motor performance and neuromuscular strength. **A**–**C** Averaged data of time to fall in the Rotarod test by mice before and after several days post-injection (dpi) with LPC in corpus callosum (cyan dots; **A**), cerebellum (green dots; **B**) or both regions (double-site injection, red dots; **C**). **D** Time to fall in the grid test by mice before and after double-site LPC injections of the same mice showing in **C**. n.s. p > 0.05, *p < 0.05; **p < 0.01; baseline (− 2 or − 5 dpi) versus LPC treatment over time (one-way ANOVA followed by Bonferroni post hoc test, n = 3–4 mice)

Motor and locomotor dysfunctions are major hallmarks of MS patients [38]. In this regard, Xie et al. [46] have recently reported a motor dysfunction due to one-site LPC injection on the corpus callosum, however, whether double-site LPC injection on both white matter tracts impairs motor function remains unknown. In this scenario, we initially examined the impact of demyelination on motor coordination using the Rotarod test by injecting single-site LPC either in callosal or cerebellar fibers (Fig. 4A, B). No significant effect was observed by injecting LPC into the corpus callosum (Fig. 4A), while a mild significant reduction in time-to-fall was found in mice injected into the cerebellum only at 7 dpi (Fig. 4B). Importantly, time-to-fall prominently declined following 7 days of LPC injection when mice were injected with LPC in both callosal and cerebellar white matter fibers (Fig. 4C) with this effect slightly diminishing after 14 or 21 days post-LPC injection but persisting within the same period (Fig. 4C). Complementarily, we assessed the neuromuscular strength using the grid test by evaluating mice’s ability to hang onto an inverted mesh [3, 23, 25]. During this task, we recorded the time it took for a mouse to fall while hanging from the inverted grid (Fig. 4D). Double-site LPC injections in both callosal and cerebellar white matter resulted in a ~ 60% decrease in time-to-fall already 5 days post-injection (Fig. 4D). This response did not fully recover even after 6 or 7 days following LPC injection, maintaining a ~ 45% reduction compared to baseline (Fig. 4D).

The above results indicate that double-site LPC-induced callosal and cerebellar demyelination causes a more intense and sustained motor dysfunction than the single LPC-injection treatment.

### Double-site LPC injection into callosal and cerebellar white matter fibers elicits cardiorespiratory impairment and hyperactive bladder

**Fig. 5:**
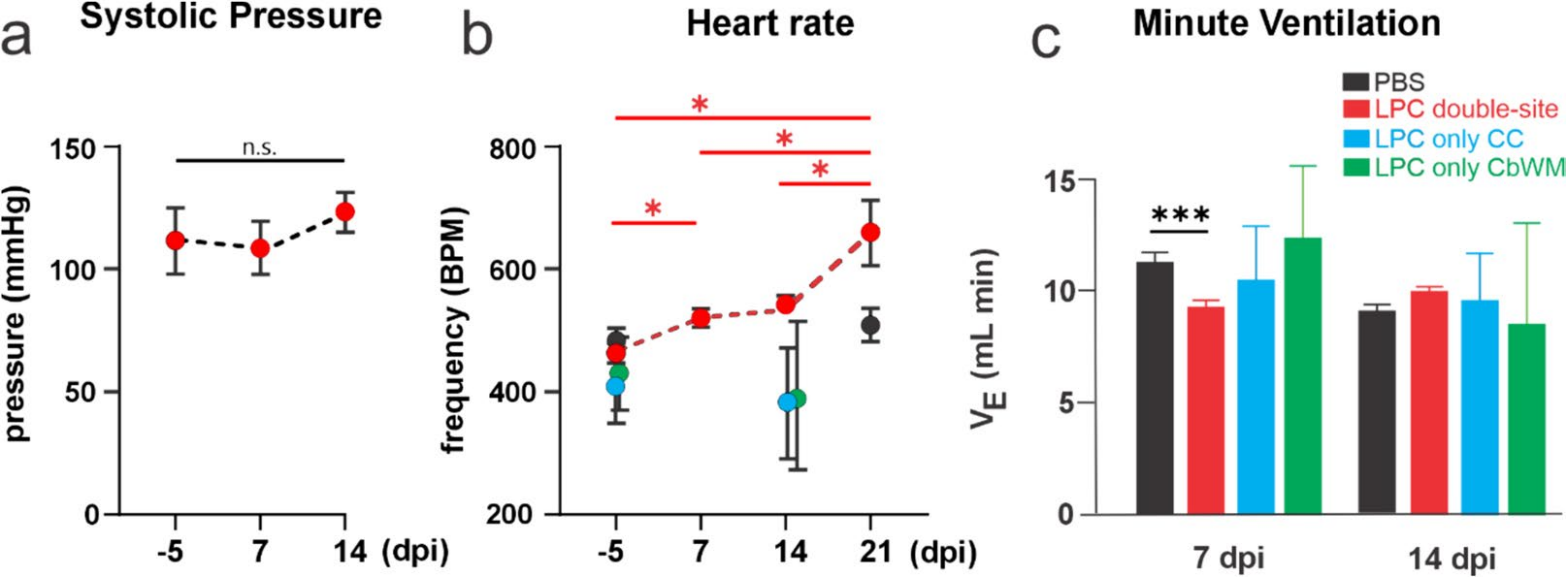
Double-site LPC injection into callosal and cerebellar white matter fibers produces tachycardia and reduced minute ventilation. **A** Averaged systolic pressure by mice before and after several days post-double-site LCP injection (dpi) in callosal and cerebellar white matter fibers. n.s., not significant p > 0.05; baseline (− 5 dpi) versus LPC treatment over time (one-way ANOVA followed by Bonferroni post hoc test, n = 4 mice). **B** Averaged frequency (heart rate, HR) of heartbeat per minute (BPM) by mice before and after several days post-double-site LCP injections (dpi) in callosal and cerebellar white matter fibers (red dots). *p < 0.05; baseline (− 5 dpi) versus LPC treatment over time (one-way ANOVA followed by Bonferroni post hoc test, n = 4 mice). LPC-injected only in callosal fibers (cyan dots, n = 2), cerebellum (green dots, n = 2) and PBS-injected mice (black dots, n = 3) showed no difference in HR at 14 and 21 dpi respect to baseline, respectively (p > 0.05, Kruskal–Wallis for PBS) (**C**) Averaged data of air volume expired per minute (V_E_) by mice following 7 or 14 days (dpi) after treated with PBS (black bars, N = 5), double-site LPC injections (red bars, N = 5 mice), LPC-injected only in callosal fibers (cyan dots, n = 3) and LPC-injected only in cerebellar white matter (green dots, n = 2). ***p < 0.001, treatment compared to control (PBS) conditions (Kruskal–Wallis test followed by a Dunns multiple comparison post hoc for PBS and LPC double-site comparison)

A commonly reported sign in MS is the autonomic dysfunction in patients, which can subsequently lead to cardiac and respiratory dysfunctions [4, 15]. Cerebellar white matter consists mostly of fibers involved in motor (i.e. corticocerebellar and spinocerebellar pathways), and autonomic (i.e. bidirectional connectivity with brainstem through peduncles) functions [17]. Considering that our animal model exhibits massive demyelination in cerebellar white matter (Figs. 1B, 2A, 3A), we investigated its impact on cardiovascular function. To achieve this, we conducted arterial pressure and heart rate measurements in mice, alongside with non-anesthetized whole-body plethysmography recordings, both before and after several days of the simultaneous LPC injection at callosal and/or cerebellar white matter fibers. We observed no significant differences in the systolic arterial pressure of animals treated by the double-site LPC injections over time (Fig. 5A). However, there was a consistent and significant increase in heart rate (HR)—or tachycardia—over time following double-site LPC injections into callosal and cerebellar white matter (Fig. 5B) while no effect on HR was found in the control group when tested 21 days after PBS injections (Fig. 5B). Moreover, 7 days after double-site LPC injections, the same animals exhibited diminished respiratory performance compared to control conditions, as measured by minute ventilation (Fig. 5C). The latter response was completely recovered following 14 days of LPC injection (Fig. 5C). Importantly, single LPC injections into the corpus callosum or cerebellum did not evoke any changes in HR (Fig. 5B) or minute ventilation (Fig. 5C).

**Fig. 6:**
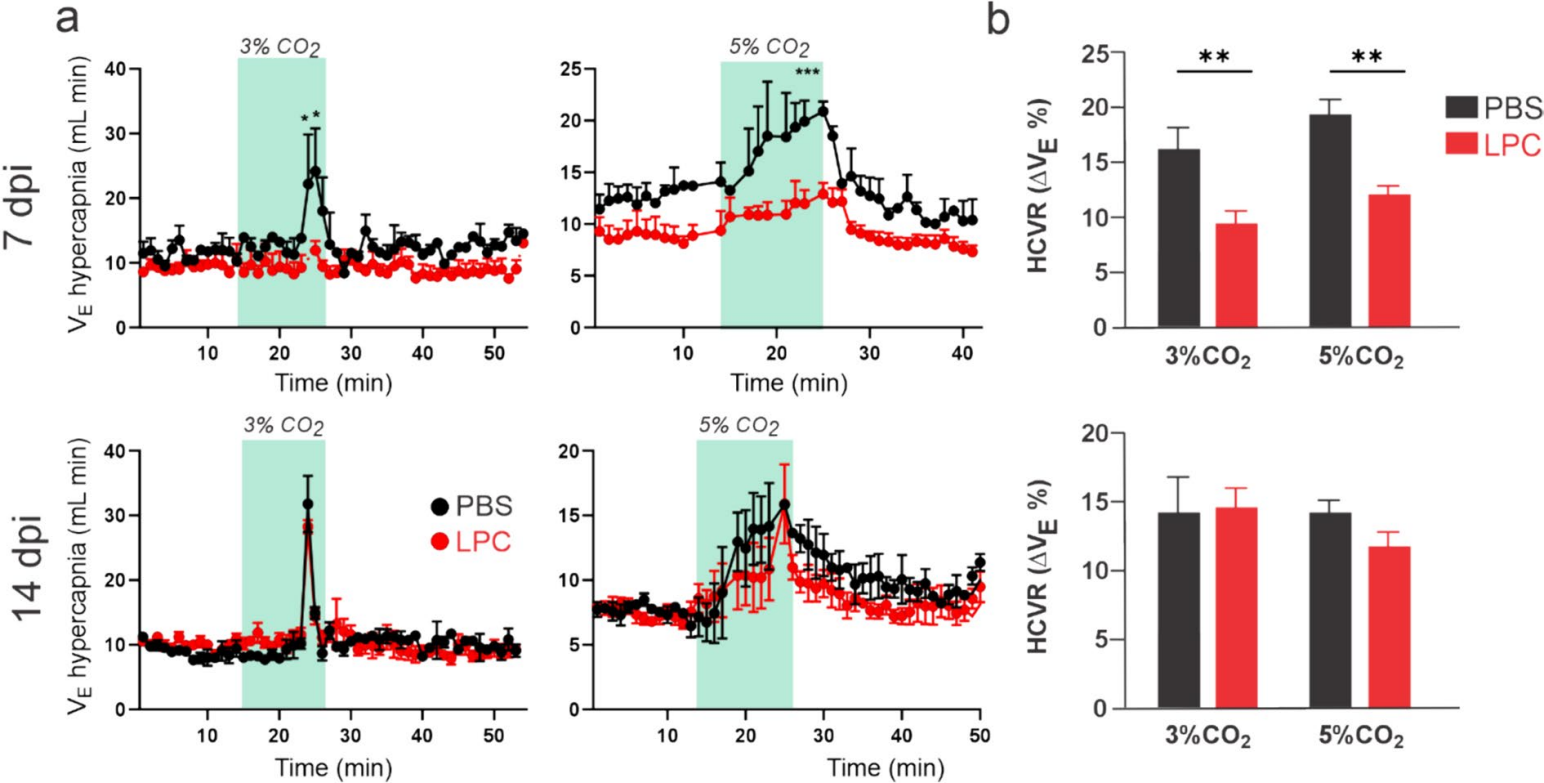
Double-site LPC injection into callosal and cerebellar white matter fibers impairs the ventilatory response to hypercapnia. **A** Averaged data of plethysmographic recordings showing minute ventilation in response to hypercapnic challenges (green blocks) of 3% (left) or 5% (right) CO_2_, by mice following 7 (top panel) or 14 (bottom panel) days post-injection (dpi) with PBS (black dots) or LPC (red dots) in the corpus callosum and cerebellar white matter. **p < 0.01; ***p < 0.001; LPC treatment compared to control (PBS) conditions (two-way ANOVA followed by Bonferroni post hoc test, n = 3–4 mice). **B** Averaged data of the hypercapnic ventilatory response (HCVR) to 3% or 5% CO_2_ by mice following 7 (top panel) or 14 (bottom panel) days post-injection (dpi) with PBS (black bars) or LPC (red bars) in the corpus callosum and cerebellar white matter **p < 0.01; LPC treatment compared to control (PBS) conditions (Kruskal–Wallis test followed by a Dunns multiple comparison post hoc, n = 3–4 mice)

To further scrutinize whether the above change could occur accompanied by changes in the respiratory function, we measured the hypercapnic ventilatory response (HVR) as an indicator of adaptive ventilatory function. For this purpose, we applied two hypercapnic stimuli, namely 3% and 5% CO_2_, during the plethysmographic recordings (Fig. 6A). PBS-treated mice exhibited a delayed and transient increase in minute ventilation in response to a 3% CO_2_ challenge 7 days post-injection (Fig. 6A). This response reflects the typical ventilatory reaction to hypercapnia expected in normal mice [8]. Importantly, double-site LPC injections in callosal and cerebellar white matter fibers decreased the minute ventilation induced by 3% CO_2_ during the same post-injection period (Fig. 6A). Furthermore, when exposed to a 5% CO_2_ challenge, the rapid and sustained increase in minute ventilation observed in PBS-treated mice was notably subdued in LPC-treated mice 7 days post-injection (Fig. 6A). Certainly, LPC treatment diminished the hypercapnic ventilatory response (HCVR) induced by both 3% and 5% CO_2_ 7 days post-injection (Fig. 6A). After 14 days post-injection, the ventilatory response to hypercapnia appeared to be similar between PBS and double site LPC-treated mice, indicating the onset of a recovery or compensatory process (Fig. 6B). Overall, these data indicate that double-site LPC injection of callosal and cerebellar white matter fibers elicits a transitory cardiorespiratory impairment.

**Fig. 7:**
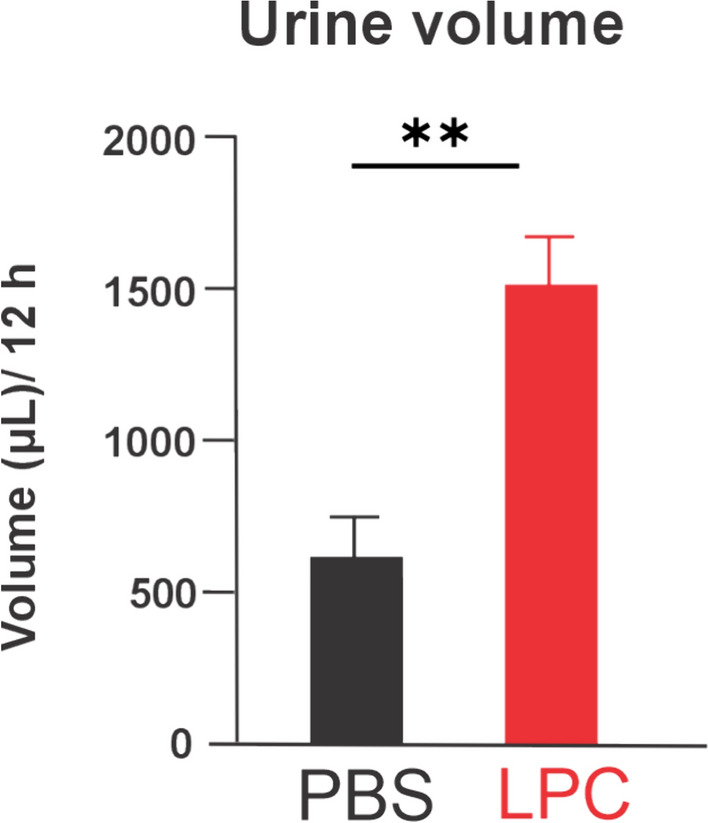
Double-site LPC injection into callosal and cerebellar white matter fibers augments the urine volume. Averaged data of urine volume measure from metabolic cages by mice following 7 days post-injection (dpi) with PBS (black bar) or LPC (red bar) in the corpus callosum and cerebellar white matter. **p < 0.01; LPC treatment compared to control (PBS) conditions. (Mann Whitney Test, n = 6 mice in control, n = 3 mice in LPC)

Hyperactive or overactive bladder is a common symptom of MS [1, 35, 36], often resulting in polyuria and/or incomplete bladder emptying [38, 44]. To investigate this, we housed control (PBS-injected) and LPC-injected mice in individual metabolic cages and measured their urine volume. We found that double-site LPC injection of callosal and cerebellar white matter fibers triplicate the urine volume production compared to the control group 7 days post-LPC injection (Fig. 7) indicating the presence of a hyperactive bladder in this animal model, at least during the demyelination window of the model (7 dpi) [32, 37].

## Discussion

MS murine models based on the single-site injection of LPC have been proven to be highly effective in reproducing relevant cellular aspects of MS-like lesions. This model allows for the identification of the remyelination stages in a highly stereotyped fashion: oligodendrocyte precursor cells (OPCs) proliferate and migrate into the lesion from 0 to 7 days post injection (dpi); then between 7 and 14 dpi, OPCs differentiate to oligodendrocytes; and remyelination of axons occurs from 14 to 30 dpi [14, 32, 37, 41]. The main advantages of this model, compared with other in vivo paradigms such as the EAE mice or cuprizone-induced lesions [42], are the follows: (i) the remyelination process is characterized by discrete and stereotyped time windows, providing an accurate time-frame for the study of mechanisms, (ii) the demyelinated lesion is a discrete area always located in the same region; (iii) since it is a focal lesion, there is no systemic immune response that could obscure cellular local effects (as in EAE for instance) [27, 42]; and (iv) the entire process (demyelination/remyelination) is relatively short for a rodent model (30 days). However, although the LPC-induced demyelination model serves as a suitable cellular model to study mechanisms of demyelination/remyelination and some neuroinflammatory signatures—with a few exceptions [47, 48], it is not accompanied by clinical-like signs. Other models partially reproduce some MS symptoms, such as motor and locomotor impairments (EAE and cuprizone models) or the autoimmune response (EAE), but still lack a more diverse and robust set of preclinical signs, to explore, for instance, putative interventions or experimental treatments.

Here, we generated an animal model exhibiting several clinical-like signs of the disease by modifying a currently stablish methodology, namely: the stereotaxic injection of LPC (see Table 1 for a summary of our major findings). By targeting two major white matter tracts, in addition to the mentioned advantages of the LPC model (i.e. mainly reproducing CNS cellular events of demyelination/remyelination), we were able to recapitulate relevant signs of the disease, such as motor and locomotor impairment, cardiac and respiratory dysfunction, and polyuria, an indicative of uronephrological dysfunction or hyperactive bladder [16].

**Table 1:** Summary and comparison of the main characteristics of LPC-injection models

Characteristic	LPC injected in CC	LPC injected in CbWM	Double-site-LPC injected in CC/CbWM
Demyelination	+	+	++
Neuroinflammatory lesions	+	+	++
Locomotor impairment	−	+	++
Neuromuscular strength impairment	n.e.	n.e.	+
Cardiovascular dysfunction	−	−	+
Ventilatory dysfunction	−	−	+
Urinary disorder	n.e.	n.e.	+

*CC* corpus callosum, *CbWM* cerebeller white matter, *n.e.* not evaluated

Motor and locomotor response impairments triggered by cuprizone-induced demyelination have been described previously [24, 28], and there are some few reports of locomotor failure induced by toxin-injections into central white matter (i.e. internal capsule, [47, 48] or CC, [46]), but to our knowledge, none of these models report any complementary signs (i.e. cardiorespiratory or bladder dysfunction). Indeed, one-site LPC injections per se—either into the corpus callosum or cerebellar white matter—usually are not associated with motor or other more complex clinical outcomes [14, 32, 37]. In this line, we found that single-site LPC injections into callosal or cerebellar white matter tract were not able to evoke major impairments on motor cardiovascular or ventilatory parameters. Then, considering that one-site LPC-injected mice do not show evident clinical-like outcomes, we decided to inject LPC in these two major white matter tracts during the same surgery under the hypothesis that we will produce diverse and evident MS-like signs in the animal. In this line, previous reports indicated that even when the cerebellum was targeted, there were not obvious motor defects (see [14]), suggesting that focal demyelinated cerebellar regions are not extensive enough to trigger significant effects, although we did find a mild reduction on locomotor performance when targeted the cerebellar fibers only (Fig. 4B). The same has been discussed regarding callosal lesions [32, 37]. Then, to overcome the possibility that the induction of too small, demyelinated areas could cover putative effects, we increased the injected toxin volume (two to four times more than reported values, 2 µL versus 0.5 to 1 µL). According to our results, the double-site LPC injection (i.e. into both callosal and cerebellar white matter fibers) induced extensive impairment functions in the animal, probably mediated by different mechanisms underlying motor/autonomic control, being thus a model suitable to study motor and cardiorespiratory mechanisms associated to demyelination. On this matter, we could speculate, that the effect on heart rate and ventilatory function (i.e. autonomic control) would be more associated with the cerebellum-brainstem communication, while the locomotor and motor dysfunction would be more related with the spinocerebellar and corticospinal functions (i.e. cortical motor neurons projections from layer 5). However, since single-LPC injections in either cerebellar or callosal fibers fails to evoke the complete cardiorespiratory and motor profile, further analysis must be done to characterize the complex responses observed in the model.

## Conclusion

Summarizing, double-site LPC-injections into the corpus callosum and cerebellar white matter induced a panel of relevant outcomes ranging from characteristic features of lesioned tissue to relevant clinical-like MS hallmarks. Thus, here we introduce a complementary preclinical mice model of MS that we hope will open new ways to deepen our knowledge on the mechanisms of demyelinated diseases and eventually the possibility of developing trials of experimental MS treatments. Although the present model provides a diverse and robust panel of MS clinical-like signs, the model lacks the characteristic immune response of MS. In this line, the EAE model must still be considered as the gold standard to study neuroinflammatory mechanisms, and therefore it could be a complementary approach to the MS model introduced here. Then, mice underwent the double-site LPC injection, showing demyelination of callosal and cerebellar white matter fibers will allow for the development of studies that use the advantages of toxin-demyelinated models combined with relevant clinical signs. This might correlate cellular mechanisms with particular clinical signs or progression time-course, but also could provide a scenario to examine the cellular mechanisms underlying common symptoms of MS such as locomotor, cardiorespiratory and bladder dysfunction.

## Data Availability

Data is available upon reasonable request to the corresponding author.

## References

[1] Akakpo W, Chartier-Kastler E, Joussain C, Denys P, Lubetzki C, Phé V. Outcomes of ileal conduit urinary diversion in patients with multiple sclerosis. Neurourol Urodyn. 2020;39(2):771–7. 10.1002/nau.24279. 31951678 10.1002/nau.24279

[2] Araos P, Prado C, Lozano M, Figueroa S, Espinoza A, Berger T, Mak TW, Jaisser F, Pacheco R, Michea L, Amador CA. Dendritic cells are crucial for cardiovascular remodeling and modulate neutrophil gelatinase-associated lipocalin expression upon mineralocorticoid receptor activation. J Hypertens. 2019;37(7):1482–92. 10.1097/HJH.0000000000002067. 31033725 10.1097/HJH.0000000000002067

[3] Bonetto A, Andersson DC, Waning DL. Assessment of muscle mass and strength in mice. Bonekey Rep. 2015;4:732. 10.1038/bonekey.2015.101. 26331011 10.1038/bonekey.2015.101 PMC4549925

[4] Briggs FBS, Hill E, Abboud H. The prevalence of hypertension in multiple sclerosis based on 37 million electronic health records from the United States. Eur J Neurol. 2021;28(2):558–66. 10.1111/ene.14557. 32981133 10.1111/ene.14557

[5] Constantinescu CS, Farooqi N, O’Brien K, Gran B. Experimental autoimmune encephalomyelitis (EAE) as a model for multiple sclerosis (MS). Br J Pharmacol. 2011;164(4):1079–106. 10.1111/j.1476-5381.2011.01302.x. 21371012 10.1111/j.1476-5381.2011.01302.x PMC3229753

[6] Criste G, Trapp B, Dutta R. Axonal loss in multiple sclerosis: causes and mechanisms. Handb Clin Neurol. 2014;122:101–13. 10.1016/B978-0-444-52001-2.00005-4. 24507515 10.1016/B978-0-444-52001-2.00005-4

[7] Deshmukh VA, Tardif V, Lyssiotis CA, Green CC, Kerman B, Kim HJ, Padmanabhan K, Swoboda JG, Ahmad I, Kondo T, Gage FH, Theofilopoulos AN, Lawson BR, Schultz PG, Lairson LL. A regenerative approach to the treatment of multiple sclerosis. Nature. 2013;502(7471):327–32. 10.1038/nature12647. 24107995 10.1038/nature12647 PMC4431622

[8] Díaz-Jara E, Pereyra K, Vicencio S, Olesen MA, Schwarz KG, Toledo C, Díaz HS, Quintanilla RA, Del Rio R. Superoxide dismutase 2 deficiency is associated with enhanced central chemoreception in mice: implications for breathing regulation. Redox Biol. 2024;69: 102992. 10.1016/j.redox.2023.102992. 38142585 10.1016/j.redox.2023.102992 PMC10788617

[9] Figueroa SM, Bertocchio J-P, Nakamura T, El-Moghrabi S, Jaisser F, Amador CA. The mineralocorticoid receptor on smooth muscle cells promotes tacrolimus-induced renal injury in mice. Pharmaceutics. 2023;15(5):1373. 10.3390/pharmaceutics15051373. 37242615 10.3390/pharmaceutics15051373 PMC10223994

[10] Franklin RJ. Remyelination of the demyelinated CNS: the case for and against transplantation of central, peripheral and olfactory glia. Brain Res Bull. 2002;57(6):827–32. 10.1016/s0361-9230(01)00765-1. 12031280 10.1016/s0361-9230(01)00765-1

[11] Franklin RJM, Ffrench-Constant C. Regenerating CNS myelin—from mechanisms to experimental medicines. Nat Rev Neurosci. 2017;18(12):753–69. 10.1038/nrn.2017.136. 29142295 10.1038/nrn.2017.136

[12] Franklin RJM, Ffrench-Constant C. Remyelination in the CNS: from biology to therapy. Nat Rev Neurosci. 2008;9(11):839–55. 10.1038/nrn2480. 18931697 10.1038/nrn2480

[13] Gharagozloo M, Mace JW, Calabresi PA. Animal models to investigate the effects of inflammation on remyelination in multiple sclerosis. Front Mol Neurosci. 2022;15: 995477. 10.3389/fnmol.2022.995477. 36407761 10.3389/fnmol.2022.995477 PMC9669474

[14] Gautier HOB, Evans KA, Volbracht K, James R, Sitnikov S, Lundgaard I, James F, Lao-Peregrin C, Reynolds R, Franklin RJM, Káradóttir RT. Neuronal activity regulates remyelination via glutamate signalling to oligodendrocyte progenitors. Nat Commun. 2015;6(1):8518. 10.1038/ncomms9518. 26439639 10.1038/ncomms9518 PMC4600759

[15] Gökaslan S, Demirbaş H, Özer Gökaslan Ç. Evaluation of cardiovascular autonomic dysfunction according to heart rate turbulence and variability in patients with relapsing remitting multiple sclerosis. Turk J Med Sci. 2020;50(2):442–7. 10.3906/sag-1912-6. 32222131 10.3906/sag-1912-6 PMC7164742

[16] Guerrero A, Visniauskas B, Cárdenas P, Figueroa SM, Vivanco J, Salinas-Parra N, Araos P, Nguyen QM, Kassan M, Amador CA, Prieto MC, Gonzalez AA. α-Ketoglutarate upregulates collecting duct (pro)renin receptor expression, tubular angiotensin II formation, and Na+ reabsorption during high glucose conditions. Front Cardiovasc Med. 2021;8: 644797. 10.3389/fcvm.2021.644797. 34179130 10.3389/fcvm.2021.644797 PMC8220822

[17] Haines DE, Dietrichs E. The cerebellum—structure and connections. In: Subramony SH, Dürr A, editors. Handbook of clinical neurology, vol. 103. Amsterdam: Elsevier; 2012. 10.1016/B978-0-444-51892-7.00001-2. 10.1016/B978-0-444-51892-7.00001-2 21827879

[18] Hall SM. The effect of injections of lysophosphatidyl choline into white matter of the adult mouse spinal cord. J Cell Sci. 1972;10(2):535–46. 10.1242/jcs.10.2.535. 5018033 10.1242/jcs.10.2.535

[19] Healy LM, Stratton JA, Kuhlmann T, Antel J. The role of glial cells in multiple sclerosis disease progression. Nat Rev Neurol. 2022;18(4):237–48. 10.1038/s41582-022-00624-x. 35190704 10.1038/s41582-022-00624-x

[20] Höftberger R, Lassmann H. Inflammatory demyelinating diseases of the central nervous system. Handb Clin Neurol. 2018;145:263–83. 10.1016/B978-0-12-802395-2.00019-5. 10.1016/B978-0-12-802395-2.00019-5 PMC7149979 28987175

[21] Humphries C. Progressive multiple sclerosis: the treatment gap. Nature. 2012;484(7393):S10. 10.1038/nature11108. 22509511 10.1038/nature11108

[22] Keough MB, Jensen SK, Yong VW. Experimental demyelination and remyelination of murine spinal cord by focal injection of lysolecithin. J Vis Exp. 2015;97:52679. 10.3791/52679. 10.3791/52679 PMC4401378 25867716

[23] Kim ST, Son HJ, Choi JH, Ji IJ, Hwang O. Vertical grid test and modified horizontal grid test are sensitive methods for evaluating motor dysfunctions in the MPTP mouse model of Parkinson’s disease. Brain Res. 2010;8(1306):176–83. 10.1016/j.brainres.2009.09.103. 10.1016/j.brainres.2009.09.103 19804765

[24] Kipp M. How to use the cuprizone model to study de- and remyelination. Int J Mol Sci. 2024;25(3):1445. 10.3390/ijms25031445. 38338724 10.3390/ijms25031445 PMC10855335

[25] Klein SM, Vykoukal J, Lechler P, Zeitler K, Gehmert S, Schreml S, Alt E, Bogdahn U, Prantl L. Noninvasive in vivo assessment of muscle impairment in the mdx mouse model—a comparison of two common wire hanging methods with two different results. J Neurosci Methods. 2012;203(2):292–7. 10.1016/j.jneumeth.2011.10.001. 22015600 10.1016/j.jneumeth.2011.10.001

[26] Kuhlmann T, Moccia M, Coetzee T, Cohen JA, Correale J, Graves J, Marrie RA, Montalban X, Yong VW, Thompson AJ, Reich DS. Multiple sclerosis progression: time for a new mechanism-driven framework. International Advisory Committee on Clinical Trials in Multiple Sclerosis. Lancet Neurol. 2023;22(1):78–88. 10.1016/S1474-4422(22)00289-7. 36410373 10.1016/S1474-4422(22)00289-7 PMC10463558

[27] Lassmann H, Bradl M. Multiple sclerosis: experimental models and reality. Acta Neuropathol. 2017;133(2):223–44. 10.1007/s00401-016-1631-4. 27766432 10.1007/s00401-016-1631-4 PMC5250666

[28] Lubrich C, Giesler P, Kipp M. Motor behavioral deficits in the cuprizone model: validity of the Rotarod test paradigm. Int J Mol Sci. 2022;23(19):11342. 10.3390/ijms231911342. 36232643 10.3390/ijms231911342 PMC9570024

[29] Maldonado PP, Guevara C, Olesen MA, Orellana JA, Quintanilla RA, Ortiz FC. Neurodegeneration in multiple sclerosis: the role of Nrf2-dependent pathways. Antioxidants. 2022. 10.3390/antiox11061146. 35740042 10.3390/antiox11061146 PMC9219619

[30] McGinley MP, Goldschmidt CH, Rae-Grant AD. Diagnosis and treatment of multiple sclerosis: a review. JAMA. 2021;325(8):765–79. 10.1001/jama.2020.26858. 33620411 10.1001/jama.2020.26858

[31] Nave K-A. Myelination and support of axonal integrity by glia. Nature. 2010;468(7321):244–52. 10.1038/nature09614. 21068833 10.1038/nature09614

[32] Ortiz FC, Habermacher C, Graciarena M, Houry P-Y, Nishiyama A, Nait Oumesmar B, Angulo MC. Neuronal activity in vivo enhances functional myelin repair. JCI Insight. 2019;5: 123434. 10.1172/jci.insight.123434. 30896448 10.1172/jci.insight.123434 PMC6538342

[33] Packer D, Fresenko EE, Harrington EP. Remyelination in animal models of multiple sclerosis: finding the elusive grail of regeneration. Front Mol Neurosci. 2023;16:1207007. 10.3389/fnmol.2023.1207007. 37448959 10.3389/fnmol.2023.1207007 PMC10338073

[34] Plemel JR, Michaels NJ, Weishaupt N, Caprariello AV, Keough MB, Rogers JA, Yukseloglu A, Lim J, Patel VV, Rawji KS, Jensen SK, Teo W, Heyne B, Whitehead SN, Stys PK, Yong VW. Mechanisms of lysophosphatidylcholine-induced demyelination: a primary lipid disrupting myelinopathy. Glia. 2017;66(2):327–47. 10.1002/glia.23245. 29068088 10.1002/glia.23245

[35] Ramasamy R, Smith PP. Animal modeling of lower urinary tract dysfunction associated with multiple sclerosis: Part I: justification of the mouse model for MS research. Neurourol Urodyn. 2021;40(4):950–7. 10.1002/nau.24649. 33719097 10.1002/nau.24649 PMC8137595

[36] Ramasamy R, Smith PP. PART 2: mouse models for multiple sclerosis research. Neurourol Urodyn. 2021;40(4):958–67. 10.1002/nau.24654. 33739481 10.1002/nau.24654 PMC8137599

[37] Sahel A, Ortiz FC, Kerninon C, Maldonado PP, Angulo MC, Nait-Oumesmar B. Alteration of synaptic connectivity of oligodendrocyte precursor cells following demyelination. Front Cell Neurosci. 2015;9:77. 10.3389/fncel.2015.00077. 25852473 10.3389/fncel.2015.00077 PMC4362325

[38] Schmidt C. Biology: a degenerative affliction. Nature. 2016;540(7631):S2–3. 10.1038/540S2a. 27902683 10.1038/540S2a

[39] Shan H-M, Maurer MA, Schwab ME. Four-parameter analysis in modified Rotarod test for detecting minor motor deficits in mice. BMC Biol. 2023;21(1):177. 10.1186/s12915-023-01679-y. 37592249 10.1186/s12915-023-01679-y PMC10433596

[40] Sheikh AM, Nagai A, Ryu JK, McLarnon JG, Kim SU, Masuda J. Lysophosphatidylcholine induces glial cell activation: role of rho kinase. Glia. 2009;57(8):898–907. 10.1002/glia.20815. 19115379 10.1002/glia.20815

[41] Trapp BD, Nave K-A. Multiple sclerosis: an immune or neurodegenerative disorder? Annu Rev Neurosci. 2008;31(1):247–69. 10.1146/annurev.neuro.30.051606.094313. 18558855 10.1146/annurev.neuro.30.051606.094313

[42] van der Star BJ, Vogel DYS, Kipp M, Puentes F, Baker D, Amor S. In vitro and in vivo models of multiple sclerosis. CNS Neurol Disord Drug Targets. 2012;11(5):570–88. 10.2174/187152712801661284. 22583443 10.2174/187152712801661284

[43] Varas R, Ortiz FC. Neuroinflammation in demyelinating diseases: oxidative stress as a modulator of glial cross-talk. Curr Pharm Des. 2019;25(45):4755–62. 10.2174/1381612825666191216125725. 31840603 10.2174/1381612825666191216125725

[44] Vecchio M, Chiaramonte R, Di Benedetto P. Management of bladder dysfunction in multiple sclerosis: a systematic review and meta-analysis of studies regarding bladder rehabilitation. Eur J Phys Rehabil Med. 2022;58(3):387–96. 10.23736/S1973-9087.22.07217-3. 35102733 10.23736/S1973-9087.22.07217-3 PMC9980558

[45] Woodruff RH, Franklin RJ. Demyelination and remyelination of the caudal cerebellar peduncle of adult rats following stereotaxic injections of lysolecithin, ethidium bromide, and complement/anti-galactocerebroside: a comparative study. Glia. 1999;25(3):216–28. 10.1002/(sici)1098-1136(19990201)25:3%3c216::aid-glia2%3e3.0.co;2-l. 9932868 10.1002/(sici)1098-1136(19990201)25:3<216::aid-glia2>3.0.co;2-l

[46] Xie Y, Chen X, Li Y, Chen S, Liu S, Yu Z, Wang W. Transforming growth factor-β1 protects against LPC-induced cognitive deficit by attenuating pyroptosis of microglia via NF-κB/ERK1/2 pathways. J Neuroinflamm. 2022;19(1):194. 10.1186/s12974-022-02557-0. 10.1186/s12974-022-02557-0 PMC9336072 35902863

[47] Yamazaki R, Ohno N, Huang JK. Acute motor deficit and subsequent remyelination-associated recovery following internal capsule demyelination in mice. J Neurochem. 2021;156(6):917–28. 10.1111/jnc.15142. 32750162 10.1111/jnc.15142 PMC8048697

[48] Yamazaki R, Osanai Y, Kouki T, Huang JK, Ohno N. Pharmacological treatment promoting remyelination enhances motor function after internal capsule demyelination in mice. Neurochem Int. 2023;164: 105505. 10.1016/j.neuint.2023.105505. 36754122 10.1016/j.neuint.2023.105505

